# Test anxiety in medical school is unrelated to academic performance but correlates with an effort/reward imbalance

**DOI:** 10.1371/journal.pone.0171220

**Published:** 2017-02-09

**Authors:** Henry Hahn, Peter Kropp, Timo Kirschstein, Gernot Rücker, Brigitte Müller-Hilke

**Affiliations:** 1 Institute of Immunology, University Medical Center Rostock, Rostock, Germany; 2 Institute of Medical Psychology and Medical Sociology, University Medical Center Rostock, Rostock, Germany; 3 Oscar Langendorff Institute of Physiology, University Medical Center Rostock, Rostock, Germany; 4 Clinic for Anesthesiology and Intensive Care Medicine, University Medical Center Rostock, Rostock, Germany; 5 Institute of Immunology and Dean of study research group, University Medical Center Rostock, Rostock, Germany; Kyoto University, JAPAN

## Abstract

**Purpose:**

During their early years at medical school, students repeatedly criticize their workload, time constraints and test associated stress. At the same time, depressiveness and anxiety among first and second year medical students are on the rise. We therefore hypothesized that test anxiety may be related to depressiveness and considered cognitive and academic performances as confounders for the former and psychosocial distress for the latter.

**Methods:**

A whole class of 200 second year students was invited to participate in the study. Anxiety as a trait, depressiveness, crystallized intelligence, verbal fluency and psychosocial distress were assessed using validated tests and questionnaires. Acute state anxiety and sympathetic stress parameters were measured in real life situations immediately before an oral and a written exam and paired tests were used to compare the individual anxieties at the various time points. Previous academic performances were self-reported, the results of the impending exams were monitored. Finally, correlations were performed to test for interrelatedness between academic performances and the various personal, cognitive and psychosocial factors.

**Results:**

Acute test anxiety did not correlate with depressiveness nor did it correlate with previous nor impending academic performances nor any of the expected confounders on academic performance. However both, depressiveness and test anxiety strongly correlated with the perceived imbalance between efforts spent and rewards received. Moreover, anxiety as a trait not only correlated with acute state anxiety before an exam but was also significantly correlated to the feeling of over-commitment.

**Conclusion:**

Depressiveness during the early years of medical school seems unrelated to test anxiety and academic performance. Instead, it strongly correlated with the psychosocial distress emanating from attending medical school and points at a perceived imbalance between efforts spent and rewards received.

## Introduction

Assessments are an integral part of medical school education and serve various purposes, among them the feedback to students and to teachers about the state of knowledge of the learners. In this respect, examinations are also a means to screen students for their aptitude and thus, the outcome of exams will have consequences. In case of failure, the students are usually allowed to re-sit an exam; however, repeated failures will cause time delays and ultimately exclusion from medical school. Assessments are therefore considered a rather stressful and anxiety-evoking part of medical education. This is particularly true for the preclinical years and it is almost exclusively during these early years that students drop out [1].

Test associated stress and anxiety might be thought to play dual roles: ideally, they encourage learning and shift the students’ academic performance along the Yerkes-Dodson curve towards a more optimal point [2]. In addition, they prepare for the stress medical students will encounter in subsequent practice and foster coping strategies [3]. Yet while some students may be motivated by the test associated anxiety and stress, for others it may be distress, that in turn was suggested to negatively influence professional development, play a role in attrition from medical school and have a devastating effect on the personal well-being [4].

Due to increasing reports on depression among medical students in their preclinical years [5, 6], we were interested, whether test anxiety is linked to depressiveness or whether there are alternative sources for depressiveness. To that extent, we searched for psychosocial stressors associated with attending medical school and borrowed from research performed on depression and burn-out at the workplace. In detail, we investigated two models, the demand-control- and the effort-reward-imbalance models [7, 8]. These were introduced by Siegrist and Karasek and imply that both, a loss of decision latitude in the face of increasing (job) demands as well as an imbalance between high efforts spent and low rewards received combined with a feeling of over-commitment lead to distress. The corresponding questionnaires–ERI (effort-reward-imbalance), OC (over commitment) and JDCQ (Job-Demand-Control-Questionnaire)—have since been translated into various languages, have been validated in a number of different work settings and have recently been adapted to the academic environment of students [9–15]. If combined with a means of evaluating depression, then depression scores consistently paralleled those of the psychosocial distress [16–19].

To investigate the relationship between anxiety, depression and academic performance for medical students, we undertook an exploratory study combining validated questionnaires with the assessment of the sympathetic stress parameters heart rate, systolic blood pressure and salivary cortisol in real life situations. We chose physiology as a subject because our students typically describe physiology as the most challenging one within the first two pre-clinical years. Moreover, education in physiology at our medical school comprises a written and an oral exam, which allowed for a direct comparison and an escalation because oral or viva voce exams were previously shown to induce particularly high test anxiety [3, 20]. Additional confounders of test associated stress and anxiety we addressed were previous academic performance, crystallized intelligence and verbal fluency. We anticipated that previous academic experiences–either success or failures–would influence acute test anxiety and that crystallized intelligence might impact on academic achievements as was recently shown for high school students [21]. Verbal fluency was expected to be of particular use in oral examinations as our professors frequently criticize the students’ lack of eloquence. Finally, we were curious whether test anxiety was linked to the impending academic achievement. The hypotheses we made were i) test anxiety is higher before oral than before written exams, ii) test anxiety and the psychosocial distress emanating from attending medical school are related to depressiveness during the early years of medical school, iii) academic performance and test anxiety impact on each other, and iv) verbal fluency alleviates test anxiety before oral exams.

## Methods

### Participant information and procedure

This explorative study involved medical students in their second pre-clinical year at the University of Rostock. One of the required courses at the beginning of the term was used to inform the whole class of 200 students about the goals of the study, before an invitation to participate was made. Written informed consent was subsequently collected during small group seminars, enrolment into the study cohort was strictly voluntary. This study was approved by the Research Ethics Committee of the University Medical Center Rostock (ref. A 2015–0052).

### Study design

The present study was carried out in cooperation with the Institute of Physiology where all students need to sit a written test, prior to attending a 7-day practical course, which in turn is paralleled by an oral exam. In case of failure, both exams can be resat within the same term. While the written retest is scheduled no earlier than 4 weeks after the first test, the oral retest is within days of the first one. Ultimate failure of either one of the exams will deny admission to the state exam at the end of the second pre-clinical year and will lead to a one-year delay. We therefore assume that the students perceived both exams to be quite important and, for many, a significant stressor.

The design of the study is summarized in Table 1. The first time point (T0) took place during the second week of term and included assessments of the psychological distress emanating from attending medical school, a test for crystallized intelligence, anxiety as a trait and baseline values for heart rate and blood pressure. Saliva for cortisol reference measurements was taken around 9 am. The second time point (T1) took place 30 minutes before the written test and included assessments of acute (test) anxiety, heart rate, blood pressure and salivary cortisol. This test was scheduled several weeks in advance and took place at 9 am. All students sat the same exam at the same time. In order to pass, 48 marks out of a maximum of 80 had to be achieved. The third time point (T2) took place 30 minutes before the oral exam and again included assessments of acute (test) anxiety, heart rate, blood pressure and salivary cortisol. This oral exam took place any time during the practical course; however, exact day and time of day for this exam were unknown to the students until 30 minutes upfront. The oral exams took place in groups of four and were pass/fail only. All students in our cohort sat their oral exam with the same professor and consequently exams were scattered throughout the day and were spread over the 7 days practical course. The fourth time point (T3) took place at the same time of day but one day after the respective oral exam and included assessments of back-to-baseline heart rate, blood pressure and salivary cortisol as well as verbal fluency and depressiveness.

**Table 1:** Study design.

T0	T1	T2	T3
second week of term	30 min before written exam	30 min before oral exam	one day after oral exam
ERI^1^, OC^2^, JDCQ^3^ MCVIT^4^			
STAI-T^5^	STAI-S^6^	STAI-S^6^	
heart rate	heart rate	heart rate	heart rate
blood pressure	blood pressure	blood pressure	blood pressure
salivary cortisol*	salivary cortisol	salivary cortisol	salivary cortisol^#^ VFT^7^, BDI-II^8^

^1^Effort-Reward-Imbalance questionnaire

^2^Over-Commitment questionnaire

^3^Job-Demand-Control questionnaire

^4^Multiple Choice Vocabulary Intelligence Test (MWT-A)

^5^State-Trait-Anxiety Inventory-Trait

^6^State-Trait-Anxiety Inventory-State

^7^VerbalFluency (letter and category) Task (RWT)

^8^Beck’s Depression Inventory II

*taken at exact time of day as T1 salivary cortisol

^#^taken at exact time of day as T2 salivary cortisol

### Data collection tools

Anxiety was assessed using the self-reported Spielberger’s state and trait anxiety inventory (STAI-S and STAI-T) [22] of which the German version was used here [23]. Stress was measured via the sympathetic activities heart rate, blood pressure and salivary cortisol. Heart rate was assessed using a CMS-50 pulse oximeter (Contec Medical Systems, Hebei Provice, People’s Republic of China), blood pressure was taken using the wrist monitor BMG 5610 (AEG, Nürnberg, Germany) and for cortisol measurements, saliva was collected into a Sarstedt-Salivette^®^ (Sarstedt, Nümbrecht, Germany) before performing an electro-chemiluminiscence immunoassay (ECLIA) on a cobas e411 analyzer (Roche, Basel, Switzerland). Psychosocial distress was assessed using two different questionnaires, which were originally designed to evaluate job contentedness. These questionnaires cover the effort-reward-imbalance, over-commitment and the job demand and control [7, 24]. They were recently rephrased to suit the academic setting of medical schools, were joined into a single questionnaire and were translated into German [9]. Crystallized intelligence was measured using a German Multiple Choice Vocabulary Intelligence Test (MWT-A) with 37 items. Each item consists of 5 words that sound very similar yet four words are purely fictional. The task at hand was to identify the non-fictional word [25]. Depressiveness was measured using Beck’s Depression Inventory (BDI-II), consisting of 21 items [26]. Verbal fluency was evaluated using the German Regensburger word fluency test (RWT), which evaluates letter as well as category fluency in four subtests. The time allocated for each subtest was 2 minutes and participants had to generate as many words as possible that fitted the specific requirements. Requirements for the letter fluency were to produce words that i) started with a specific letter, ii) alternated in their starting letter between two specific ones, iii) belonged to a specific category ("first names", "animals", "hobbies", "professions" or "foods") and that iv) alternated between two categories [27]. Academic performance was evaluated twofold, by collecting the written test results (physiology only) and the self-reported overall grades from the previous term (covering physiology, anatomy, biochemistry and medical psychology). S1 Table summarizes the published reliability coefficients (Cronbach’s alpha) to substantiate the credibility of the various questionnaires and tests used. Further psychometric information can be obtained within the references cited above.

### Data analyses

Prior to statistical evaluation, all data sets were tested for Gaussian distribution performing Shapiro-Wilk tests. Because some data sets did not follow Gaussian distribution, only non-parametric methods were used for further analyses.

Friedman Tests (nonparametric repeated measures ANOVA) were performed to follow anxiety and the sympathetic stress parameters longitudinally over the various time points. As for the power analysis, we used previous publications to approximate the size of the expected effect [28, 29]. Assuming an alpha error of 0.05 and a power of 0.8 yielded group sizes between n = 8 and n = 29.

Spearman Rank Correlation Analyses were used to determine correlation coefficients and these were corrected for multiple comparisons according to Bonferroni. For post hoc power analyses, raw p values <0.05 were considered statistically significant. Acute test anxiety at T1 or T2 correlated the strongest with an effort-reward-imbalance and the respective correlation coefficients of 0.4967 and 0.5637 yielded a power of 0.96 and 0.99, respectively.

Reliability of test results was evaluated calculating Cronbach’s alpha. All statistical tests were carried out using either IBM SPSS Statistics 20 or Microsoft Excel. Power analysis was performed using G*Power version 3.1.9.2.

## Results

### Preliminary statistics

Out of a total of 200 second year medical students, 72 enrolled in this study and 48 completed it. Reasons for dropping out were the postponement of either written (n = 7) or oral exam (n = 3), a leave of absence after enrolment (n = 5), sitting the oral exam with an alternate examiner (n = 4) or the decision to discontinue without giving any reasons (n = 5). In summary, the mean age of the study population was 21.9 (±2.7) years, 15/48 (33%) were male, and they achieved a mean 55.4 (±8.9) out of a total of 80 possible scores. These data are comparable to those of the remaining students of the class who did not participate.

### Test anxiety is higher before oral than before written exams

**Fig 1:**
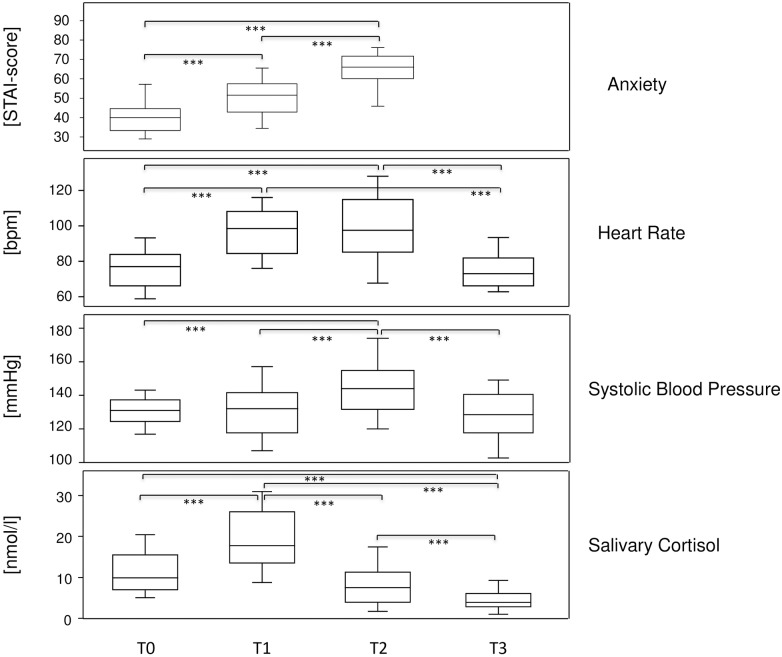
Test anxiety is higher before oral than before written exams. Box plots show medians for self-reported state anxiety as well as measured heart rate, systolic blood pressure and salivary cortisol levels immediately before the written test (T1), the oral exam (T2) and at the reference time points at the beginning (T0) and at the end (T3) of the term, respectively. Note, that for the assessment of salivary cortisol, T0 and T3 were sampled at the exact time of day as T1 and T2, respectively. The boxes represent medians as well as upper and lower quartiles and the whiskers indicate 90^th^ and 10^th^ percentiles, respectively. *** indicate significant results (P <0.001) from Friedman tests (nonparametric repeated measures ANOVA).

We also aimed to confirm that test anxiety can be measured in real life situations and that sympathetic stress parameters parallel the self-reported anxiety. To that extent, anxiety was assessed longitudinally at various time points throughout the term—at the beginning (T0), immediately before written (T1) and oral exam (T2) and one day after the oral exam (T3) (see Table 1). Various parameters were collected to quantify anxiety, among them the self-completed STAI-T questionnaire at T0 to assess trait anxiety and STAI-S at T1 and T2 to evaluate acute state anxiety before an exam, here used synonymously with test anxiety. Sympathetic indicators of stress were heart rate, blood pressure and salivary cortisol levels. At each time point, all the study participants were sampled for all the parameters indicated and the resultant data are summarized in the S2 Table. After matching the data, they were evaluated, performing nonparametric repeated measures ANOVA. Fig 1 summarizes the results and shows that the self-reported anxiety levels were lowest at the beginning of the term, increased significantly before the written and even more so before the oral exam (upper panel). Heart rate followed a comparable pattern whereby highest values were measured at T1 and T2 before written and oral exam respectively, and were back to T0 levels one day after the oral exam (T3). Interestingly, the medians for systolic blood pressure at T0 and T1 were comparable and thus suggested the absence of an increase in blood pressure before the written test. However, at close inspection these medians represent three different groups of students, those whose blood pressure hardly changed at all (N = 23), increased (N = 14) or decreased (N = 11) more than one standard deviation from the values at term beginning (S2 Table). This differential response pattern was observed for blood pressure, only. Salivary cortisol levels were also significantly increased before the exams. Yet because salivary cortisol levels follow a diurnal rhythm, T0 as baseline for T1 was taken at the exact time of day as T1 and T3 was taken at the exact time of day as T2. Note that the written test took place at 9 am when salivary cortisol is close to its daily peak while the oral exams were scattered throughout the day. Absolute values for salivary cortisol were therefore higher at T0 than at T3 and likewise at T1 compared to T2. The mean increases before oral and written exams were 2.4-fold and 2.1-fold, respectively (Fig 1). Our data thus confirm that test anxiety can be measured in real life situations, that sympathetic stress parameters parallel the self-reported anxiety and that oral exams are correlated with higher anxiety levels than written ones.

### Depressiveness correlated with the psychosocial distress emanating from attending medical school but not with test anxiety

We here aimed to identify factors that influence depressiveness as well as test anxiety. To that extent, we assessed depressiveness and cognitive performances on the one hand and psychosocial distress associated with medical school on the other. For the former we used a standardized depression inventory (BDI) as well as tests for verbal fluency (RWT) and crystallized intelligence (MWT-A). For the latter we used validated questionnaires covering the perceived psychological demands (JDCQ-pd), decision latitude (JDCQ-dl) and social support (JDCQ-ss), an over-commitment (OC) and the balance between efforts spent and rewards received (ERI). The original data points are presented in S3 and S4 Tables, respectively. Reliability of the questionnaires was confirmed by Cronbach’s alphas of 0.87 (BDI), 0.77 (JDCQ), 0.75 (ERI+OC), 0.91 (STAI-T), 0.93 (STAI-S_T1_) and 0.92 (STAI-S_T2_), respectively. The outcomes of these assessments are summarized in Table 2.

**Table 2:** Test results and cognitive functions of study participants.

	Mean ± SD	Range
Effort-Reward-Imbalance (ratio)	0.99 ± 0.21	(0.64–1.37)
Over-Commitment	17.0 ± 3.3	(11–24)
Job-Demand-Control Questionnaire		
Psychological Demand	16.0 ± 1.7	(11–19)
Decision Latitude	15.4 ± 1.7	(11–21)
Social Support	10.4 ± 2.3	(7–17)
STAI-T	40.9 ± 10.0	(23–68)
STAI-S _T1_	50.9 ± 10.6	(26–72)
STAI-S _T2_	63.3 ± 10.4	(35–78)
Beck’s Depression Inventory-II	10.7 ± 6.3	(1–25)
Previous Term Grades	2.9 ± 0.8	(2–5)
Multiple-Choice Vocabulary Test (MWT-A)	32.0 ± 2.2	(25–35)
Verbal Fluency Task (RWT)		
task 1 (letter fluency)*	31.4 ± 23.5	(1–93)
task 2 (alternating letters)*	35.1 ± 24.4	(2–96)
task 3 (category fluency)*	48.2 ± 26.3	(2–97)
task 4 (alternating categories)*	46.0 ± 24.9	(4–99)

*percent range of control cohort

To quantify the relationship between possible confounders, depressiveness and test anxiety, we performed Spearman Rank correlation analyses. To that extent, we concentrated on the self-reported anxieties as assessed via the STAI questionnaires at T0, T1 and T2 and excluded the sympathetic stress parameters because of the irregularities observed for blood pressure and the diurnal rhythm of salivary cortisol. Finally we included all primary data into one correlation analysis and the results thereof are depicted in Table 3. Significant correlations were between test anxiety (at T1 and T2) and an imbalance between performed efforts and rewards received (ERI) as well between test anxiety and the perceived psychological demands associated with attending medical school (JDCQpd). All of these correlations were stronger for test anxiety before the oral (T2) than before the written (T1) exam. Interestingly, while test anxiety neither correlated with depressiveness nor with any of the cognitive performances, anxiety as a trait significantly correlated with depressiveness. However, the strongest correlation was between depressiveness and the perceived imbalance between efforts spent and rewards received. Of note, previous term grades and the results of the written test correlate very well and thus serve as a confirmation for the consistency of our data set.

**Table 3:** Correlations between trait anxiety, acute test anxiety, cognitive functions and psychosocial distress.

	STAI-S_T1_	STAI-S_T2_	Beck’s Depression Inventory	Over Commitment	JDCQ-psychological demand	Effort-Reward-Imbalance	Previous Term Grades	Written TestResults	Verbal Fluency Task 4	Multiple Choice Vocabulary Test (MWT-A)
STAI-T	0.43440.16–0.650.0012	0.50330.25–0.690.0003	0.50470.24–0.700.0003	0.73340.56–0.85<0.0001	0.48650.23–0.680.0005	0.56860.33–0.74<0.0001	0.49760.24–0.690.0004	n.s.	-0.4486–0.66 to -0.180.0014	n.s.
STAI-S_T1_		0.58020.35–0.75<0.0001	n.s.	n.s.	0.42230.15–0.640.0028	0.49670.24–0.690.0003	n.s.	n.s.	n.s.	n.s.
STAI-S_T2_			n.s.	0.44710.18–0.650.0014	0.47650.21–0.680.0006	0.56370.33–0.74<0.0001	n.s.	n.s.	n.s.	n.s.
Beck’s Depression Inventory				n.s.	0.45400.18–0.660.0015	0.60730.38–0.77<0.0001	n.s.	n.s.	n.s.	n.s.
Over Commitment					0.41770.14–0.630.0031	0.48140.22–0.680.0005	-0.4619–0.67 to -0.190.0007	n.s.	n.s.	n.s.
JDCQ-psychological demand						0.54760.30–0.72<0.0001	n.s.	n.s.	n.s.	n.s.
Effort-Reward-Imbalance							*-0*.*3997–0*.*62 to -0*.*120*.*0054*	n.s.	n.s.	n.s.
Previous TermGrades								0.47560.21–0.680.0005	n.s.	n.s.
Written TestResults									n.s.	n.s.
Verbal FluencyTask 4										n.s.

Spearman Rank Correlation Analysis. Above the diagonal: correlation coefficient ρ, corresponding 95% confidence interval and P-value. Italic formatting: statistical significance will get lost upon correction for multiple comparisons (according to Bonferroni). n.s.: not significant at all.

### Academic performance and test anxiety in medical school did not correlate with each other

Academic performance in medical school was assessed either as self-reported mean grades obtained in the previous term (covering physiology, anatomy, biochemistry and medical psychology) or as the score from the written exam that was part of this study and covered physiology, only. The oral exam was pass or fail only and results thus allowed no further discrimination between low and high performance. Because low grades in the German system correspond to excellent performance, these data were inverted to have them match the written test scores in direction. Importantly, we did not observe any correlation between test anxiety and subsequent or previous academic performance or test anxiety and depressiveness. However, previous term grades correlated with trait anxiety as well as a perceived over-commitment (Table 3).

### Verbal fluency was not related to test anxiety but was inversely correlated with anxiety as trait

Trait anxiety correlated best with a feeling of over-commitment (ρ = 0.73) followed by an imbalance between high efforts spent and low rewards received (ρ = 0.57), scores for depressiveness (ρ = 0.51) and the perceived psychological demand associated with attending medical school (ρ = 0.49) (Table 3). Moreover, verbal fluency correlated negatively with trait anxiety (ρ = -0.45). Even though we here assessed letter as well as category fluency, it was only subtask 4—testing for alternating categories–that was negatively correlated with trait anxiety.

## Discussion

We here show that self-reported test anxiety in medical students is a state of hyperarousal that was corroborated by elevated sympathetic activity assessed as salivary cortisol levels, heart rate and blood pressure. We were intrigued that the median increase in blood pressure before the written test was only minor but are inclined to speculate that the fixed time and date of this exam allowed for some self-medication to control for the hyperarousal. Our speculation is supported by the significant increase in blood pressure before the spontaneously scheduled oral exam.

The present study shows that test anxiety is higher before oral than before written exams and thus confirms our first hypothesis. We can only speculate that it is the fear of individual failure in the presence of fellow students and face to face with the professor in combination with the importance attributed to the exam that aggravates oral exams [30].

Moreover, we show that test anxiety did not correlate with depressiveness. This finding disproves part of our second hypothesis and shows that the increased levels of depressiveness observed among first and second year medical students seem unrelated to test anxiety [9, 5]. Depressiveness also did not correlate with academic performance suggesting that, neither failure at an exam nor poor results are related to depressiveness. Instead, depressiveness as well as trait- and acute test anxiety strongly correlated with the psychosocial distress emanating from attending medical school, confirming the second part of our second hypothesis. Indeed, we here show for the first time, that the context between psychosocial distress, depressiveness and anxiety–which has been described for the work place—also holds true for medical students [16–19]. While this context corroborates the internal consistency of our results, it shows the dilemma medical schools are facing: Reducing test anxiety may not necessarily be the declared goal as both, students and faculty at our university agree that exposure to stressful situations fosters the development of coping strategies. Indeed, when sitting the statewide exam at the end of their second year, the students participating in the present study achieved their highest test scores in physiology (data available at https://www.impp.de/internet/de/archiv.html). However, preventing depressiveness among students is a global concern [4]. While the workload of the students cannot be reduced without impairing the teaching, it is the esteem question within the effort-reward-imbalance questionnaire that points to a self-reported deficit among the students. The outmost challenge for medical schools is therefore to foster an environment that is characterized by esteem and at the same time supports maximal academic performance.

Interestingly, our data disprove our third hypothesis and provided no evidence, that test anxiety and academic performance–be it previous or impending—impact on each other. Our results are thus in contrast to a previous publication on university antecedents that reported a small but significant negative impact of test anxiety on academic performance [31]. At best, the fact that test anxiety did not relate to poor performance at our faculty may be considered assurance that hyperarousal did not reach a stage at which students’ performance was adversely affected [32, 2]. Alternatively, these previously reported correlations may not apply to medical students at all or our number of participants is too small and therefore obscures small effect sizes.

Our results also disprove our fourth hypothesis, that verbal fluency alleviates test anxiety before oral exams. However, we did find a correlation between anxiety as a trait and verbal fluency. Fluency tasks have regularly been used in research and non-clinical groups alike to assess executive control ability [33]. These executive controls comprise a set of functions considered to regulate thoughts and direct behavior towards a general goal. However, the observation that verbal fluency performance correlated with acute state anxiety during a life-threatening situation prompted the suggestion that executive control capacity also regulates emotions during very stressful situations [29]. As of yet, there are conflicting reports as to whether executive control ability is primarily expressed in performance in the letter as opposed to the category fluency task [33]. We are therefore intrigued by our observation that only the alternating category fluency capacity was negatively correlated to trait anxiety. Future research thus needs to investigate in more detail how trait anxiety and executive functioning are interrelated and whether either one can be used to predict success or failure in medical school. Interestingly, the capacity to regulate emotions as part of emotional intelligence has been suggested to predict effective communication and interpersonal sensitivity, which in turn are predictive of successful doctor-patient relationships and therapeutic outcomes [34, 35].

Our small sample size is the clear limitation to our study. With only 48 participants, we are bound to miss small effects. For instance, we did not observe any significant differences between the sexes with respect to test anxiety, even though age, sex and being a medical student have been shown to confound anxiety as a trait [4]. However, we consider our study an explorative pilot for further research with larger cohorts and possibly a more restricted set of variables in order to perform structure equation models.

A side effect of this study was the identification of possible predictors for success in medical school. As of yet, entrance qualifications like high school grades and medical college admission test results are widely accepted as predictors for academic achievements in medical school [36–38]. These predictors were therefore not analyzed any further in the present study. Instead we here show that elevated trait anxiety may be predictive of academic failure. Indeed, it was recently summarized that not only intelligence but also non-intellective factors impact on academic achievements, even though these findings applied to compulsory and tertiary education [31]. We were in fact intrigued that crystallized intelligence did not correlate with students’ grades in our study. However, the predictive power of intelligence tends to drop as students progress and advance to higher levels of formal education—particularly at medical school, where selection procedures assure homogeneous cognitive ability levels [21].

## Supporting information

S1 Table Reliability of the various questionnaires and tests used. (DOCX)

S2 Table Anxiety and sympathetic stress parameters at the various time points. (DOCX)

S3 Table Demographics, academic achievements and cognitive accomplishments. (DOCX)

S4 Table Psychosocial stressors and depressiveness. (DOCX)
