# Supplementary material for: Test anxiety in medical school is unrelated to academic performance but correlates with an effort/reward imbalance
Source: PLoS One. 2017 Feb 9;12(2):e0171220. doi: 10.1371/journal.pone.0171220 (PMC5300107; doi:10.1371/journal.pone.0171220)
Supplement: S1 Table — (DOCX) [file pone.0171220.s001.docx]

**S1 Table.** Reliability of the various questionnaires and tests used

Questionnaire/Test Cronbach’s alpha

Effort-Reward-Imbalance Questionnaire

Subscale Effort 0.74

Subscale Reward 0.79

Subscale Over-Commitment 0.79

Job-Demand-Control Questionnaire 0.73-0.74

Multiple Choice Vocabulary Intelligence Test 0.87

State-Trait-Anxiety Inventory

Subscale State 0.9-0.94

Subscale Trait 0.88-0.94

Verbal Fluency (letter and category) Task (RWT) 0.72-0.89

Beck’s Depression Inventory 0.84-0.91
