# Supplementary material for: Test anxiety in medical school is unrelated to academic performance but correlates with an effort/reward imbalance
Source: PLoS One. 2017 Feb 9;12(2):e0171220. doi: 10.1371/journal.pone.0171220 (PMC5300107; doi:10.1371/journal.pone.0171220)
Supplement: S2 Table — (DOCX) [file pone.0171220.s002.docx]

**S2 Table. Anxiety and sympathetic stress parameters at the various time points**

STAI-T STAI-S systolic blood pressure [mmHg] heart rate [bpm] salivary cortisol [nmol/l]

participant T0 T1 T2 T0 T1 T2 T3 T0 T1 T2 T3 T0 T1 T2 T3

1 35 44 60 131 140 165 130 66 119 78 15.6 19 5.49 15.6

2 36 52 60 136 109 145 153 83 116 116 109 14.5 17.5 3.74 2.43

3 32 51 65 135 166 152 137 85 115 146 83 11.9 7.96 17.4 6.21

4 51 37 60 121 132 144 113 67 80 87 63 5.34 16.2 9.29 3.98

5 40 48 60 127 132 134 119 79 117 97 95 12.7 15.4 10.4 10.2

6 41 58 70 131 147 120 100 60 94 104 69 7.28 22.7 14.6 2.21

7 54 56 71 126 155 183 121 76 85 104 86 7.35 7.51 1 1

8 29 46 46 125 127 144 103 79 97 110 72 12.1 32.4 5.99 2.71

9 56 61.5 75 124 104 144 110 79 82 128 80 5.13 9.1 11.4 3.91

10 57 67 72 136 92 156 135 80 98 108 87 21.5 28.6 15.3 13.5

11 41 58 76 134 146 154 143 57 98 115 82 12.4 17.6 17.7 7.51

12 43.5 66 65 140 157 174 137 58 84 90 49 21.8 28.3

13 34 51 53 107 126 129 105 67 76 85 78 5.31 13.9 11.8 4.04

14 40 42 42.5 138 132 115 123 63 90 91 82 9.9 17.9 13.8 4.73

15 33 61 63 125 144 126 117 75 99 107 62 10.6 28.2 18.9 7.53

16 48 53 63 136 136 139 97 59 101 85 76 20.3 22.3 5.19 3.78

17 59 72 65 123 117 120 120 78 84 72 88 5.34 9.19 7.53 5.34

18 40.5 55 59 117 122 139 104 96 110 93 76 9.04 36.3 7.57 9.16

19 30 46 67 126 158 174 150 67 76 82 65 8.66 7.59 5.36 3.35

20 37 36 71 136 104 145 120 93 100 90 82 12.1 21.4 6.86 1

21 36 41 61 142 107 186 91 64 83 83 6.89 15.7 2.7 7.04

22 40 53 67 130 143 155 149 66 122 128 71 22.5 30.8 1.74 5.38

23 45 53 70 142 133 146 128 89 120 128 82 19.7 22.4 8.31 7.65

24 66 53 74 145 159 149 144 79 106 62 71 7.71 14.2 1.78 1.64

25 42 54 67 133 140 155 130 77 72 98 72 5.18 9.23 11.4 3.4

26 60 67 67 130 141 140 137 87 100 120 80 15.1 25.3 3.81 2.8

27 29 48.5 59 142 134 137 117 58 85 90 64 12.8 27 2.45 1.86

28 38 38 64 140 142 161 137 62 100 64 64 2.67 21.4 7.81 3.15

29 46 50 62 132 107 118 114 72 91 86 59 25.2 9.95 9.68

30 32 33 45 130 124 140 100 91 99 109 83 6.01 8.82 4.98 2.74

31 27 59 72 116 116 154 126 65 80 93 60 6.31 12.4 15.8 2.89

32 23 26 35 128 117 141 132 58 112 89 78 13.8 16.8 10.3 3.92

33 44 63 75 107 119 147 124 64 100 110 80 4.73 11.8 21.3 2.19

34 41 55 63 143 129 156 145 94 103 101 93 8.42 27.9 5.26 3.48

35 38 54 75 122 127 128 124 74 89 62 68 15.5 17.4 3.4 3.19

36 34 64 67 136 133 179 150 84 109 110 72 20.4 41 12.1 8.82

37 41 55 66 123 113 131 132 75 97 115 100 20.5 16.1 11.1 4.29

38 42 34 78 135 135 133 145 88 116 120 64 16.2 20.6 9.28 1

39 30 35 46 123 126 129 121 78 94 68 69 19.9 14.6 4.09 2.72

40 42 39.5 49 140 170 142 150 80 80 83 67 15.7 26.4 1 8.13

41 30 61 70 118 121 166 140 77 106 110 91 17.1 39.1 6.26 1

42 32 44 54 146 112 150 148 86 73 96 70 7.87 18.2 10.4 5.88

43 49 49 77 129 152 130 141 76 110 132 64 8.47 11.7 19 6.22

44 42 68 78 176 122 131 144 94 100 130 98 1 5.82 1.65 1

45 41 51 67 106 136 156 121 71 109 65 66 7.61 30.5 3.08 2.32

46 31 43 56 125 134 155 123 70 116 76 73 7.85 13.2 6.13 2.89

47 36 32 39 144 108 111 129 97 90 79 63 4.79 16 1.72 4.87

48 68 59 74 132 119 142 130 78 106 116 73 8.95 20.4 6.88 5.84

Black denotes less than one SD change from T0, red denotes more than 1 SD increase from T0, green denotes more than 1 SD decrease from T0.
