# Supplementary material for: Test anxiety in medical school is unrelated to academic performance but correlates with an effort/reward imbalance
Source: PLoS One. 2017 Feb 9;12(2):e0171220. doi: 10.1371/journal.pone.0171220 (PMC5300107; doi:10.1371/journal.pone.0171220)
Supplement: S3 Table — (DOCX) [file pone.0171220.s003.docx]

**S3 Table. Demographics, academic achievements and cognitive accomplishments**

Verbal Fluency Tasks [rank percentages]

| participant | age [years] | sex | written test results [%] | oral exam passed | previous academic achievements | MWT-A | Task 1 | Task 2 | Task 3 | Task 4 |
| --- | --- | --- | --- | --- | --- | --- | --- | --- | --- | --- |
| 1 | 22 | f | 53.75 | y | 3.0 | 31 | 58 | 23 | 15 | 4 |
| 2 | 20 | f | 71.25 | y | 2.5 | 34 | 70 | 24 | 74 | 98 |
| 3 | 28 | m | 87.50 | y | 2.5 | 33 | 38 | 23 | 18 | 38 |
| 4 | 20 | f | 66.25 | y | 3.0 | 35 | 19 | 48 | 24 | 30 |
| 5 | 20 | f | 85.00 | y | 3.0 | 35 | 20 | 60 | 51 | 74 |
| 6 | 28 | m | 75.00 | y | 2.0 | 31 | 37 | 36 | 44 | 62 |
| 7 | 21 | f | 60.00 | y | 3.0 | 31 | 11 | 2 | 70 | 38 |
| 8 | 21 | f | 68.75 | y | 2.0 | 34 | 16 | 48 | 69 | 74 |
| 9 | 28 | f | 53.75 | y | 5.0 | 34 | 44 | 40 | 35 | 30 |
| 10 | 28 | f | 63.75 | y | 3.0 | 33 | 45 | 14 | 86 | 37 |
| 11 | 28 | f | 67.50 | y | 4.0 | 34 | 31 | 60 | 56 | 30 |
| 12 | 26 | m | 53.75 | n | 3.0 | 32 | 8 | 13 | 12 | 27 |
| 13 | 21 | f | 40.00 | y | 5.0 | 32 | 69 | 76 | 89 | 88 |
| 14 | 27 | m | 83.75 | y | 2.0 | 34 | 32 | 41 | 63 | 27 |
| 15 | 20 | f | 56.25 | y | 3.0 | 32 | 50 | 96 | 71 | 99 |
| 16 | 25 | f | 46.25 | y | 3.5 | 28 | 55 | 36 | 32 | 4 |
| 17 | 21 | m | 71.25 | y | 2.5 | 35 | 11 | 24 | <1 | 17 |
| 18 | 24 | f | 82.50 | y | 2.0 | 32 | 53 | 84 | 59 | 62 |
| 19 | 22 | m | 76.25 | y | 2.0 | 33 | 15 | 24 | 49 | 56 |
| 20 | 21 | f | 57.50 | y |  | 32 | 11 | 7 | 65 | 49 |
| 21 | 20 | m | 70.00 | y | 2.0 | 30 | 13 | 10 | 31 | 56 |
| 22 | 20 | f | 71.25 | y | 3.0 | 31 | 9 | 14 | 5 | 32 |
| 23 | 19 | f | 70.00 | y | 3.0 | 31 | 42 | 48 | 56 | 61 |
| 24 | 19 | f | 77.50 | y | 3.0 | 32 | 15 | 9 | 74 | 27 |
| 25 | 23 | m | 80.00 | y | 3.0 | 25 | 45 | 56 | 10 | 32 |
| 26 | 21 | f | 75.00 | y | 2.0 | 28 | 24 | 9 | 41 | 30 |
| 27 | 20 | f | 83.75 | y | 2.5 | 31 | 6 | 19 | 2 | 30 |
| 28 | 20 | m | 77.50 | y | 2.0 | 33 | 6 | 20 | 61 | 56 |
| 29 | 20 | f | 77.50 | y | 3.0 | 28 | 4 | 32 | 7 | 17 |
| 30 | 23 | f | 70.00 | y | 2.0 | 34 | 6 | 14 | 66 | 81 |
| 31 | 21 | f | 65.00 | y | 2.5 | 30 | 17 | 36 | 32 | 61 |
| 32 | 20 | m | 80.00 | y | 2.5 | 35 | 44 | 29 | 53 | 83 |
| 33 | 21 | f | 68.75 | n | 5.0 | 32 | 51 | 79 | 69 | 49 |
| 34 | 21 | f | 77.50 | y | 3.0 | 31 | 9 | 36 | 43 | 17 |
| 35 | 22 | f | 73.75 | y | 3.0 | 30 | 6 | 7 | 32 | 27 |
| 36 | 20 | f | 52.00 | y | 3.0 | 30 | 50 | 27 | 61 | 56 |
| 37 | 21 | m | 43.75 | y | 3.0 | 33 | 1 | 10 | 75 | 49 |
| 38 | 21 | f | 58.75 | y | 5.0 | 29 | 32 | 40 | 97 | 38 |
| 39 | 20 | f | 72.50 | y | 2.5 | 34 | 7 | 7 | 5 | 9 |
| 40 | 20 | m | 68.75 | y | 3.5 | 32 | 21 | 10 | 12 | 48 |
| 41 | 21 | f | 81.25 | y | 2.0 | 31 | 21 | 32 | 60 | 48 |
| 42 | 21 | m | 65.00 | y | 2.0 | 32 | 15 | 36 | 68 | 27 |
| 43 | 20 | f | 82.50 | y | 3.0 | 32 | 20 | 11 | 11 | 4 |
| 44 | 22 | f | 63.75 | y | 2.7 | 33 | 69 | 56 | 75 | 74 |
| 45 | 22 | f | 78.75 | y | 2.0 | 34 | 93 | 60 | 33 | 67 |
| 46 | 20 | f | 75.00 | y | 2.0 | 30 | 93 | 27 | 59 | 79 |
| 47 | 18 | m | 70.00 | y | 2.8 | 35 | 64 | 82 | >99 | 81 |
| 48 | 24 | m | 73.75 | y | 4.0 | 34 | 30 | 89 | 97 | 27 |
