# Supplementary material for: Test anxiety in medical school is unrelated to academic performance but correlates with an effort/reward imbalance
Source: PLoS One. 2017 Feb 9;12(2):e0171220. doi: 10.1371/journal.pone.0171220 (PMC5300107; doi:10.1371/journal.pone.0171220)
Supplement: S4 Table — (DOCX) [file pone.0171220.s004.docx]

**S4 Table. Psychosocial stressors and depressiveness**

| \| participant \| BDI-II \| JDCQ psychological demands \| JDCQ decision latitude \| JDCQ social support \| Effort Reward Imbalance \| Over Commitment \| \| --- \| --- \| --- \| --- \| --- \| --- \| --- \| \| 1 \| 6 \| 13 \| 14 \| 13 \| 0.74 \| 17 \| \| 2 \| 9 \| 15 \| 17 \| 9 \| 0.75 \| 18 \| \| 3 \| 9 \| 16 \| 21 \| 14 \| 1.00 \| 12 \| \| 4 \| 5 \| 11 \| 17 \| 11 \| 0.64 \| 21 \| \| 5 \|  \| 16 \| 16 \| 11 \| 0.82 \| 13 \| \| 6 \| 1 \| 18 \| 15 \| 9 \| 0.97 \| 18 \| \| 7 \| 9 \| 16 \| 15 \| 11 \| 1.30 \| 21 \| \| 8 \| 9 \| 14 \| 15 \| 8 \| 1.01 \| 14 \| \| 9 \| 8 \| 17 \| 16 \| 10 \| 1.17 \| 24 \| \| 10 \| 19 \| 18 \| 15 \| 8 \| 1.23 \| 21 \| \| 11 \| 4 \| 17 \| 17 \| 8 \| 0.86 \| 21 \| \| 12 \|  \| 18 \| 15 \| 8 \| 1.17 \| 20 \| \| 13 \| 2 \| 16 \| 16 \| 11 \| 0.89 \| 16 \| \| 14 \| 4 \| 15 \| 15 \| 11 \| 0.68 \| 15 \| \| 15 \| 20 \| 17 \| 15 \| 9 \| 1.05 \| 15 \| \| 16 \| 17 \| 17 \| 13 \| 15 \| 1.05 \| 20 \| \| 17 \| 17 \| 18 \| 18 \| 15 \| 1.06 \| 19 \| \| 18 \| 18 \| 16 \| 16 \| 11 \| 0.93 \| 16 \| \| 19 \| 6 \| 16 \| 11 \| 10 \| 0.78 \| 18 \| \| 20 \| 7 \| 16 \| 14 \| 8 \| 1.05 \| 20 \| \| 21 \| 10 \| 14 \| 15 \| 11 \| 0.68 \| 16 \| \| 22 \| 17 \| 18 \| 13 \| 9 \| 1.35 \| 19 \| \| 23 \| 7 \| 14 \| 15 \| 13 \| 1.05 \| 17 \| \| 24 \| 16 \| 18 \| 15 \| 12 \| 1.06 \| 24 \| \| 25 \| 9 \| 17 \| 14 \| 11 \| 0.93 \| 17 \| \| 26 \| 16 \| 17 \| 16 \| 8 \| 1.35 \| 19 \| \| 27 \| 5 \| 15 \| 16 \| 12 \| 0.78 \| 13 \| \| 28 \| 15 \| 17 \| 15 \| 9 \| 0.97 \| 19 \| \| 29 \| 18 \| 17 \| 15 \| 12 \| 1.11 \| 19 \| \| 30 \| 3 \| 15 \| 17 \| 17 \| 0.68 \| 12 \| \| 31 \| 3 \| 13.5 \| 14 \| 7 \| 0.91 \| 12 \| \| 32 \| 3 \| 12 \| 12 \| 8 \| 0.72 \| 12 \| \| 33 \| 14 \| 17 \| 17 \| 11 \| 1.30 \| 16 \| \| 34 \| 6 \| 16 \| 14 \| 9 \| 1.11 \| 18 \| \| 35 \| 20 \| 15 \| 14 \| 8 \| 1.35 \| 16 \| \| 36 \| 8 \| 16 \| 16 \| 8 \| 0.89 \| 16 \| \| 37 \| 14 \| 15 \| 16 \| 10 \| 0.97 \| 17 \| \| 38 \| 25 \| 19 \| 16 \| 10 \| 1.11 \| 19 \| \| 39 \| 5 \| 16 \| 15 \| 8 \| 0.93 \| 12 \| \| 40 \| 15 \| 18 \| 17 \| 13 \| 1.00 \| 15 \| \| 41 \| 8 \| 18 \| 14 \| 9 \| 0.64 \| 11 \| \| 42 \| 8 \| 13 \| 14 \| 8 \| 0.78 \| 12 \| \| 43 \| 24 \| 18 \| 16 \| 12 \| 1.23 \| 20 \| \| 44 \| 8 \| 18 \| 14 \| 14 \| 1.37 \| 14.5 \| \| 45 \| 13 \| 14 \| 17 \| 13 \| 0.85 \| 13 \| \| 46 \| 9 \| 16 \| 18 \| 9 \| 1.23 \| 18 \| \| 47 \| 4 \| 16 \| 15 \| 7 \| 0.85 \| 18 \| \| 48 \| 21 \| 16 \| 19 \| 13 \| 1.35 \| 23 \| |  |  |  |  |  |  |
| --- | --- | --- | --- | --- | --- | --- | --- | --- | --- | --- | --- | --- | --- | --- | --- | --- | --- | --- | --- | --- | --- | --- | --- | --- | --- | --- | --- | --- | --- | --- | --- | --- | --- | --- | --- | --- | --- | --- | --- | --- | --- | --- | --- | --- | --- | --- | --- | --- | --- | --- | --- | --- | --- | --- | --- | --- | --- | --- | --- | --- | --- | --- | --- | --- | --- | --- | --- | --- | --- | --- | --- | --- | --- | --- | --- | --- | --- | --- | --- | --- | --- | --- | --- | --- | --- | --- | --- | --- | --- | --- | --- | --- | --- | --- | --- | --- | --- | --- | --- | --- | --- | --- | --- | --- | --- | --- | --- | --- | --- | --- | --- | --- | --- | --- | --- | --- | --- | --- | --- | --- | --- | --- | --- | --- | --- | --- | --- | --- | --- | --- | --- | --- | --- | --- | --- | --- | --- | --- | --- | --- | --- | --- | --- | --- | --- | --- | --- | --- | --- | --- | --- | --- | --- | --- | --- | --- | --- | --- | --- | --- | --- | --- | --- | --- | --- | --- | --- | --- | --- | --- | --- | --- | --- | --- | --- | --- | --- | --- | --- | --- | --- | --- | --- | --- | --- | --- | --- | --- | --- | --- | --- | --- | --- | --- | --- | --- | --- | --- | --- | --- | --- | --- | --- | --- | --- | --- | --- | --- | --- | --- | --- | --- | --- | --- | --- | --- | --- | --- | --- | --- | --- | --- | --- | --- | --- | --- | --- | --- | --- | --- | --- | --- | --- | --- | --- | --- | --- | --- | --- | --- | --- | --- | --- | --- | --- | --- | --- | --- | --- | --- | --- | --- | --- | --- | --- | --- | --- | --- | --- | --- | --- | --- | --- | --- | --- | --- | --- | --- | --- | --- | --- | --- | --- | --- | --- | --- | --- | --- | --- | --- | --- | --- | --- | --- | --- | --- | --- | --- | --- | --- | --- | --- | --- | --- | --- | --- | --- | --- | --- | --- | --- | --- | --- | --- | --- | --- | --- | --- | --- | --- | --- | --- | --- | --- | --- | --- | --- | --- | --- | --- | --- | --- | --- | --- | --- | --- | --- | --- | --- | --- | --- | --- | --- | --- | --- | --- | --- | --- | --- | --- | --- | --- | --- | --- | --- | --- | --- | --- | --- |
|  |  |  |  |  |  |  |
